# Stable Redox-Cycling Nitroxide Tempol Has Antifungal and Immune-Modulatory Properties

**DOI:** 10.3389/fmicb.2019.01843

**Published:** 2019-08-20

**Authors:** Ava Hosseinzadeh, Marios Stylianou, José Pedro Lopes, Daniel C. Müller, André Häggman, Sandra Holmberg, Christian Grumaz, Anders Johansson, Kai Sohn, Christoph Dieterich, Constantin F. Urban

**Affiliations:** ^1^Department of Clinical Microbiology, Umeå University, Umeå, Sweden; ^2^Umeå Centre for Microbial Research, Umeå University, Umeå, Sweden; ^3^Laboratory for Molecular Infection Medicine Sweden, Umeå University, Umeå, Sweden; ^4^Department of Molecular Biotechnology, Fraunhofer Institute for Interfacial Engineering and Biotechnology, Stuttgart, Germany; ^5^Department of Internal Medicine III, Klaus Tschira Institute for Integrative Computational Cardiology, University Hospital Heidelberg, Heidelberg, Germany

**Keywords:** antifungal activity, redox active, immunomodulators, candidiasis, *Candida albicans*, *Candida glabrata*

## Abstract

Invasive mycoses remain underdiagnosed and difficult to treat. Hospitalized individuals with compromised immunity increase in number and constitute the main risk group for severe fungal infections. Current antifungal therapy is hampered by slow and insensitive diagnostics and frequent toxic side effects of standard antifungal drugs. Identification of new antifungal compounds with high efficacy and low toxicity is therefore urgently required. We investigated the antifungal activity of tempol, a cell-permeable nitroxide. To narrow down possible mode of action we used RNA-seq technology and metabolomics to probe for pathways specifically disrupted in the human fungal pathogen *Candida albicans* due to tempol administration. We found genes upregulated which are involved in iron homeostasis, mitochondrial stress, steroid synthesis, and amino acid metabolism. In an *ex vivo* whole blood infection, tempol treatment reduced *C. albicans* colony forming units and at the same time increased the release of pro-inflammatory cytokines, such as interleukin 8 (IL-8, monocyte chemoattractant protein-1, and macrophage migration inhibitory factor). In a systemic mouse model, tempol was partially protective with a significant reduction of fungal burden in the kidneys of infected animals during infection onset. The results obtained propose tempol as a promising new antifungal compound and open new opportunities for the future development of novel therapies.

## Introduction

In recent decades, susceptibility to infection due to reduced host immunity is the most common driving force for the rise of incidences of severe fungal infections. Among others, hematologic malignancies, organ failure followed by transplantation or disruption of the microflora by antibacterial therapy create niches for opportunistic fungal organisms to shift from benign colonization to invasive infection with hazardous consequences for the host. Fungemia has an annual prevalence of 300,000 cases with a mortality rate of 30–50% (Zilberberg et al., 2008; Arendrup, 2010) and emerging multi-resistant strains, such as of *Candida auris* responsible for several nosocomial outbreaks in recent years, increase clinical concern (Quindos et al., 2018).

Current drugs against mycoses have a limited set of target structures in the fungal cell, are frequently inefficient or have toxic side effects. A common trend of the past years has been that pharmaceutical companies withdrew from development of new antimicrobial agents due to reservations concerning the market share, manifold risks during development as well as cost and time effort through clinical phases (Spellberg et al., 2004; Edwards et al., 2018). However, the rising incidences of severe mycoses, emergence of resistant strains, and the adverse side effects of current antifungal therapies illustrate the urgent need for new options.

To initiate identification of potential therapy options, we reasoned that approaches used against plant pathogenic fungi might also be useful in animals and humans. Nitroxide derivatives are examples of good drug candidates which are radical scavengers and superoxide dismutase mimetics and have been shown to be active against several fungal plant pathogens, *Botrytis cinerea*, *Fusarium culmorum*, *Phytophthora cactorum*, and *Rhizoctonia solani* (Zakrzewski and Krawczyk, 2011). Moreover, nitroxide compounds termed diazeniumdiolates were synergistic against *Candida albicans* in combination with azoles (Mcelhaney-Feser et al., 1998). A previous study has suggested targeting antioxidant pathways by pharmacological inhibition of thioredoxin in fungal pathogens as a successful therapy for experimental corneal infection in mice (Leal et al., 2012). Given that both, ROS scavenging and blockage of antioxidants, seem to be successful approaches against diverse fungal pathogens, we hypothesized that clearance could rely on disturbance of redox-balances in the fungal cell. We therefore set out to challenge the approach to use redox-interfering compounds as potential antifungal agent against systemic infection caused by human fungal pathogens. We used redox-cycling nitroxide 4-hydroxy-TEMPO (tempol) which is a stable and cell-permeable nitroxide (Davis et al., 2011). In addition, tempol has a versatile functional scope as anti-inflammatory and antineoplastic molecule (Wilcox, 2010), but antifungal activity has not been described. The low molecular weight and low cytotoxicity (Wilcox, 2010) render tempol to a favorable candidate molecule for repurposing as potential antifungal therapy. In the following, we explored the antifungal activity of tempol against *C. albicans* investigated pathways engaged in the treated fungal cells in order to narrow down a possible mode of action and tested the compound in a systemic candidiasis model.

## Results

### Fungicidal Activity of Tempol Assessed in *Candida* Species

**FIGURE 1:**
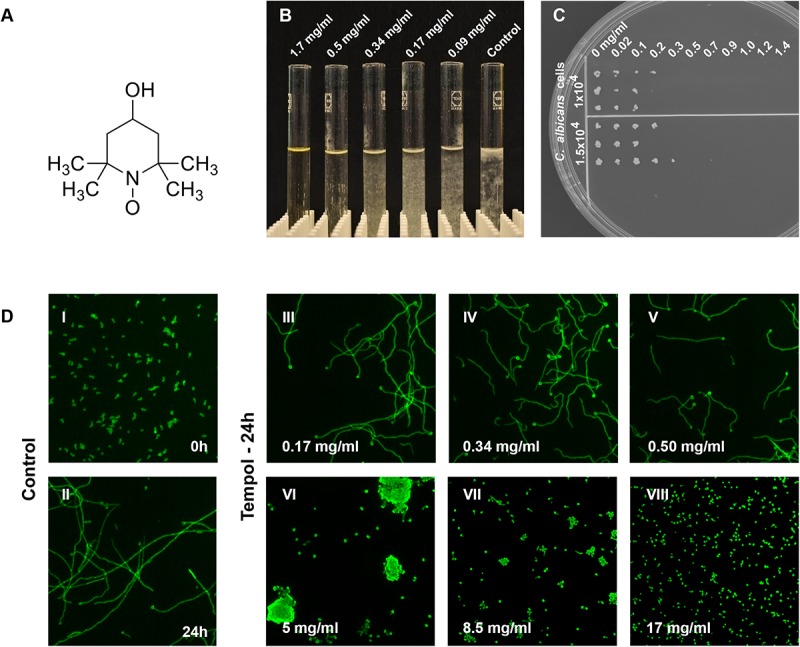
Inhibition of *C. albicans* growth by tempol results in a potent fungicidal effect. **(A)** Tempol (4-hydroxy-TEMPO) is a membrane-permeable nitroxide radical scavenger. **(B)** Macroscopic view of the dose-dependent effect of tempol on *C. albicans* growth. **(C)** To determine MFC, *C. albicans* cells (1.5 × 10^–4^ and 1 × 10^–4^ cells) were incubated with increasing concentrations of tempol (0–1.4 mg/ml) for 24 h and challenged cells were then transferred to YEPD plates using a multi-comb device. Plates were incubated for 24 h at 30°C. **(D)** Tempol caused stress phenotype – shortened and curled hyphae – in *C. albicans* after 24 h incubation at concentrations equal to or below 5 mg/ml, higher concentrations of tempol lead to growth arrest of *C. albicans*. Micrographs were taken using a fluorescence automated microscope (HCA-Cellomics ArrayScan VTI, Thermo Scientific) using *C. albicans* cells fixed with 2% paraformaldehyde and stained with 0.1% calcofluor white (CFW; Sigma–Aldrich) (Stylianou et al., 2015).

Since interference with antioxidant pathways in fungal pathogens was suggested as therapy option (Leal et al., 2012) we wanted to investigate whether radical scavenger tempol (Figure 1A) could hamper *C. albicans* growth. For this purpose, we cultured *C. albicans* in the presence of increasing tempol concentrations ranging from 0.05 to 17 mg/ml. Indeed, tempol affected fungal cell viability (Table 1) and phenotypic appearance (Figures 1B–D). We defined the inhibitory concentration (IC) as the tempol concentration at which metabolic activity was reduced over 90% compared to controls (Table 1 and Figure 1C). Replica plating showed that tempol has fungicidal activity for *C. albicans* type strain SC5314 at 0.5 mg/ml (Table 1 and Figure 1C). Interestingly, tempol was also active against a *Candida glabrata* type strain ATCC 90030 (Supplementary Figure S1 and Supplementary Table S1). To asses a broader antifungal activity we subjected several clinical *C. albicans* and *C. glabrata* isolates to tempol treatment (Supplementary Table S2). While the IC_50_ values were comparable, in average, the IC_90_ values were slightly higher for the clinical isolates than for the type strains. Only one clinical isolate of *C. glabrata* showed a more than threefold higher IC_90_ than the *C. glabrata* type strain (Supplementary Table S2). In summary, tempol exerts antifungal activity against *Candida* type strains and clinical isolates retrieved from blood cultures.

**TABLE 1:** *In vitro* activity of tempol against *C. albicans* SC5314 assessed by 3 different methods.

**IC and MFC of tempol against *C. albicans***			
**Tempol**	**ATP**	**OD_530_ (nm)**	**Agar-spot assay**
Tested concentration	0.05–17	0.05–17	–
range (mg/ml)			
IC_90_^a^	0.68	0.5	–
IC_50_^b^	0.15	0.3	–
MFC^c^	–	–	0.5

*^*a,b*^Inhibitory concentration (IC) was determined as the lowest concentration of tempol that inhibited fungal growth at ≥90% ^*a*^IC_90_ or ≥50% ^*b*^IC_50_; Data from three independent experiments. ^*c*^Minimum fungicidal concentration (MFC) was determined as the lowest concentration of tempol that killed at least 99.9% of the initial inoculum. Data from three independent experiments.*

### Tempol Is Efficient at the Initial Phase of Infection in a Mouse Model of Systemic Candidiasis

To test the *in vivo* anti-*Candida* activity of tempol we infected two groups of mice intravenously (i.v.) with *C. albicans*. One group was intraperitoneally (i.p.) treated with approximately 182 μg tempol/g mouse in 100 μl phosphate buffered saline (PBS) once daily for 3 days while the other group was sham-treated with an equal volume of PBS. We chose this dose in compliance with a very detailed *in vivo* pharmacokinetic study on tempol in mice reporting peak concentrations of 8.1 mM tempol in plasma (Davis et al., 2011), a concentration exceeding efficacy concentrations determined with *in vitro* assays. The anti-*Candida* effect of tempol *in vivo* was monitored using the following parameters: mouse weight and temperature, colony forming unit (CFU) in kidney, and blood biochemistry testing.

**FIGURE 2:**
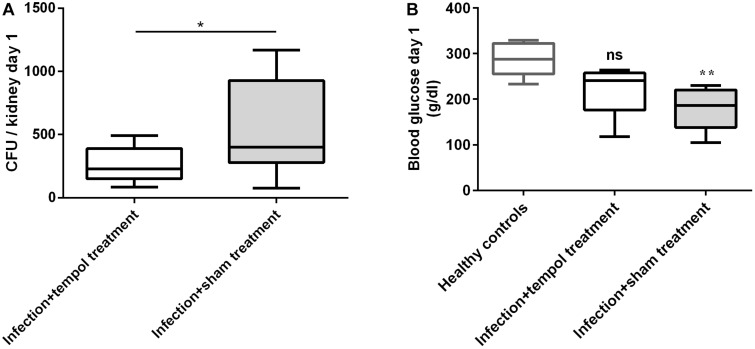
Tempol reduces fungal burden and improves fitness of mice at day 1 *p.i*. **(A)** *C. albicans* CFU from kidneys of mice infected i.v. with 2.6 × 10^6^ cells/ml of *C. albicans* are shown. Mice were injected i.p. with tempol in PBS (1.6 mg/g of mouse/day) or with PBS only. Tempol treatment reduced the fungal burden of kidneys. **(B)** Blood glucose was reduced in sham-treated mice when compared to uninfected mice, suggesting improper nutrition of affected animals. Tempol-treated animals showed intermediate blood glucose levels suggesting an ameliorated fitness state. **(A,B)** Bars represent mean ± *SD* with ^*^p < 0.05; ^∗∗^*p* < 0.01; ns = not significant.

Fungal load, described as CFU per kidney at day 1 post-infection (*p.i.*) was significantly lower in tempol-treated mice (Figure 2A). In agreement, average temperature of infected mice was slightly elevated on day 1, when compared to infected mice treated with tempol (Supplementary Figure S2A). At day 3, the fungal load, temperature, and weight between the groups were similar (Supplementary Figures S2A–C) despite an ameliorated fitness as shown by increased glucose levels in the blood of tempol-treated animals (Figure 2B). Tempol had a promising transient effect during initial phases, while efficacy was decreasing during later time points of infection probably due to the short half-life of nitroxides *in vivo* (Davis et al., 2011). In summary, tempol could transiently reduce fungal burden in kidneys of mice systemically infected with *C. albicans.* Further studies testing different types of nitroxides and formulations are required to improve the therapeutic window.

### Tempol Exerts Antifungal Activity by Modulating Fungal Metabolic Pathways and Host Immune Responses

**FIGURE 3:**
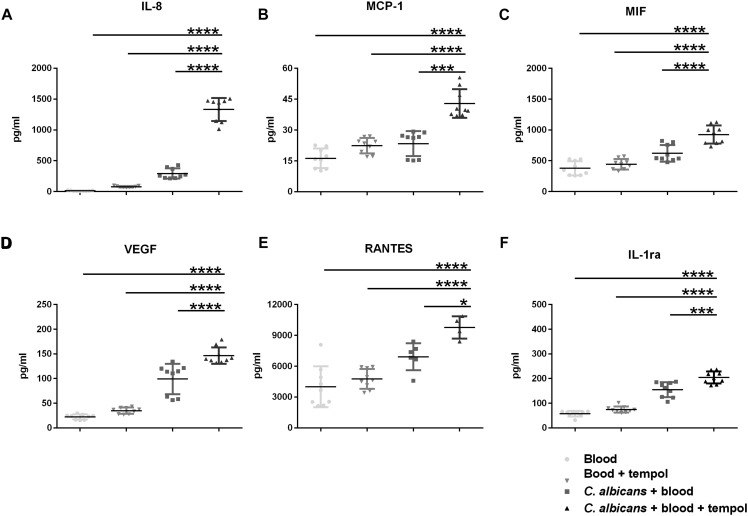
Tempol induces a strong immune response upon infection of *C. albicans* in whole blood. Human heparinized blood samples from three healthy volunteers were stimulated for 24 h with *C. albicans* (2 × 10^5^ cells/ml) in presence or absence of 0.516 mg/ml tempol. The concentrations of **(A)** IL-8, **(B)** MCP-1, **(C)** MIF, **(D)** VEGF, **(E)** RANTES, and **(F)** IL-1Ra were measured in cell supernatants using Bio-plex cytokine array. **(A–F)** Bars represent mean ± SD with ^*^p < 0.05; ^∗∗∗^*p* < 0.001; ^****^p < 0.0001.

The improved glucose levels in tempol-treated animals led us to hypothesize that tempol could have a dual role acting on both, the fungal and the host side. To address this, we decided to assess the impact of tempol on cytokine signaling after *C. albicans* infection. We infected whole human, heparinized blood with *C. albicans*, incubated overnight, and measured cytokine levels in separated plasma. Surprisingly, *C. albicans*-infected blood treated with tempol showed a substantial increase in several inflammatory cytokines compared to infected but untreated as well as uninfected but treated samples (Figure 3). The detected cytokines are involved in early innate responses and include granulocyte chemokine interleukin-8 (IL-8), monocyte chemotactic protein-1 (MCP-1), and macrophage migration inhibitory factor (MIF) (Figures 3A–C), as well as the pro-inflammatory cytokine vascular endothelium growth factor (VEGF) (Figure 3D), the T-cell-associated cytokine RANTES (Figure 3E), and the receptor antagonist (IL-1ra) which is crucial for modulation of inflammation during infections (Figure 3F). Importantly, our studies allowed a direct link between administration of tempol and a regulation of pro-inflammatory responses in plasma upon *C. albicans* infection *ex vivo*.

**FIGURE 4:**
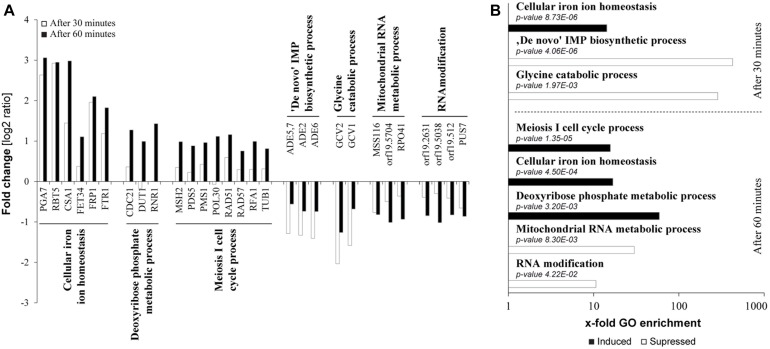
Transcriptional dysregulation induced by tempol targets iron homeostasis and mitochondria. **(A)** Fold changes of mRNA expression in *C*. *albicans* after 30 or 60 min of tempol-treated (0.344 mg/ml) compared to untreated *C. albicans* samples. Presented genes are representative, differentially regulated genes from affected pathways as assessed according to a GO term enrichment analysis. **(B)** Samples described in **(A)** were analyzed using gene ontology enrichment of affected processes in *C*. *albicans* in response to tempol. Corresponding sets of up- and down-regulated genes were mapped to biological processes using the “GO term finder” at the *Candida* Genome database (CGD). X-fold enrichment is calculated as the ratio of percentages of the cluster frequency of tested gene set and the cluster frequency of genomic background.

**FIGURE 5:**
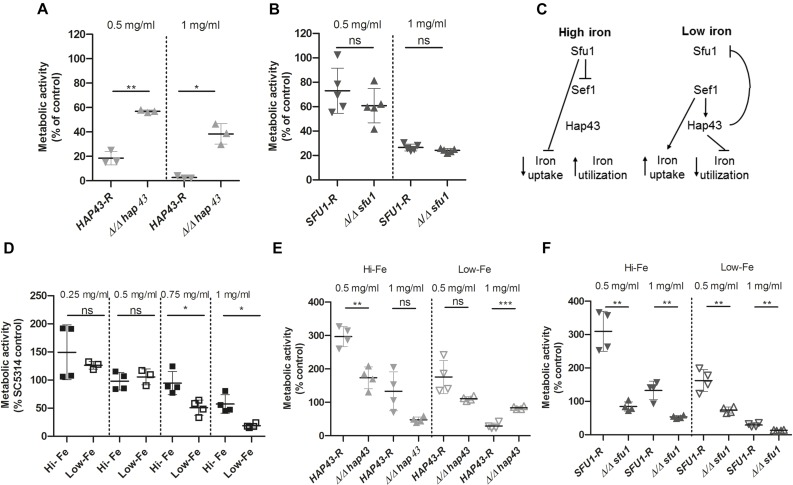
Anti-*Candida* activity of tempol depends on expression of *HAP43*. **(A)** Δ*/*Δ *hap43* knockout mutant is less susceptible to tempol. Fungal viability was assessed using ATP quantification in three biological replicates with five technical replicates each [*n* = 3(5)]. Fungal strains were grown overnight at 30°C and subcultured in SC medium for 4 h before being seeded in a 96-well plate containing RPMI medium with two different tempol concentrations (0.5 and 1 mg/ml) and incubated for 24 h. A Δ*/*Δ *hap43* knockout mutant was compared to a revertant strain derived from the knockout mutant. **(B)** Δ*/*Δ *sfu1* knockout mutant is more susceptible to tempol. Similar experimental setup as above in five biological replicates with four technical replicates each [*n* = 5(4)]. **(C)** Simplified scheme of iron homeostasis regulation in *C. albicans* adapted from Chen et al. (2011). **(D)** *C. albicans* is less susceptible to tempol under high iron than under low iron conditions. High and low iron conditions in YEPD were achieved as described in the section “Materials and Methods.” Pre-cultured cells were subsequently seeded in a 96-well plate containing either high or low iron medium with different tempol concentrations and incubated for 24 h. Fungal viability was assessed using ATP quantification in four biological replicates with four technical replicates each [*n* = 4(4)]. **(E,F)** *HAP43* but not *SFU1* promotes antifungal effect of tempol. Similarly as above we assessed viability of **(E)** *Δhap43* and **(F)** *Δsfu1* homozygous deletion mutants in comparison with respective revertant strains under high and low iron conditions in four biological replicates with four technical replicates each [*n* = 4(4)]. ATP was quantified after 24 h incubation with two different tempol concentrations (0.5 and 1 mg/ml). Scatter plots represent mean ± SD with ^*^p < 0.05; ^∗∗^*p* < 0.01; ^∗∗∗^*p* < 0.001; ns = not significant.

In an effort to characterize potential modes of action of tempol in the dysregulation of fungal homeostasis we studied transcriptome changes of *C. albicans by* performing RNA-Seq (Figures 4A,B and Supplementary Table S3). The average Pearson correlation for the duplicates based on the RPKM values was 0.92 and confirmation by qPCR has been published previously for similar analyses (Grumaz et al., 2013). After 30 min incubation in the presence of tempol (0.344 mg/ml) 10 genes (0.13%) and after 60 min incubation 82 genes out of a total of 6668 genes (1.2%) were identified as differentially regulated. Gene ontology (GO) term enrichment of the differentially expressed genes revealed that tempol treatment induced expression of genes involved in cellular iron homeostasis, in cell cycle and division, and in metabolic processes of deoxyribose phosphate after 30 and 60 min of incubation (Figures 4A,B). Suppressed genes upon tempol treatment were involved in processes of *de novo* purine biosynthesis, glycine catabolic processes, mitochondrial RNA metabolic processes, and RNA modification after 30 and 60 min of incubation (Figures 4A,B). Interestingly, it has been shown that mitochondrial function and iron homeostasis are intertwined (Xu et al., 2014). Dysregulation of iron homeostasis affects mitochondrial physiology. Both vacuolar iron transporter Ccc1p and mitochondrial iron transporter Mrs4p are required for maintenance of mitochondrial function, since numerous mitochondrial proteins depend on iron ions as essential co-factor. Another clear link became evident. After 60 min of tempol treatment 22 genes were differentially expressed (16 upregulated and 6 downregulated) which are regulated by the transcription factor Hap43p (Supplementary Figure S3). This factor has been shown to be essential for iron-responsive regulation and virulence (Chen et al., 2011; Hsu et al., 2011). Notably, Hap43p also regulates genes involved in mitochondrial physiology and stress adaption, such as *MSS*116, coding a putative DEAD-box helicase (De Silva et al., 2017) involved in splicing of mitochondrial introns (Singh et al., 2011) and orf 19.2631, coding a putative protein involved in tRNA modification (Figure 4A and Supplementary Tables S3C,D). Since several of the deregulated genes are controlled by iron-responsive regulator Hap43p, we decided to test the effect of tempol in a *hap43* null mutant strain (Figure 5A). At both tempol concentrations tested the Δ*hap43* homozygous knockout strain was not more susceptible to tempol treatment, but instead more resistant against tempol. This suggests that tempol-induced stress responses in *C. albicans* rather contribute to detrimental effects which are consequently absent in the Δ*hap43* strain and clearly indicates that tempol treatment induces an interlinked dysregulation of iron homeostasis and mitochondrial function. To test dysregulation of iron homeostasis further as potential target of tempol we tested another iron regulator the transcription factor Sfu1p. A homozygous deletion mutant Δ*sfu1* was more susceptible to tempol treatment (Figure 5B) as a revertant strain. This makes sense, since Sfu1p is a repressor of Sef1p the activator of Hap43p and of iron uptake genes (Figure 5C). Thus, tempol exerts its toxic effect under conditions where *HAP43* is expressed. To investigate how iron levels influence tempol activity we incubated wild-type *C. albicans* in high iron and low iron media compared to our previous experiments conducted norm-iron conditions (RPMI and SC media). In consistence, *C. albicans* was more susceptible to tempol treatment under low iron than under high iron conditions (Figure 5D). This difference was not evident at lower tempol concentrations (up to 0.5 mg/ml), but increased at higher tempol concentrations (up to 1 mg/ml).

Next, we tested the susceptibility of Δ*hap43* and Δ*sfu1* deletion strains toward tempol under high and low iron conditions. As expected, the Δ*hap43* was more resistant to tempol under low iron conditions, but slightly more or equally susceptible under high iron conditions compared to the revertant strain (Figure 5E). This is consistent to the previous notion that *HAP43* expression is required for full toxicity of tempol. *HAP43* is not expressed under high iron conditions, since its activator Sef1p is repressed by Sfu1p (Figure 5C). In agreement with this, the Δ*sfu1* mutant is slightly more susceptible to tempol both under high and low iron conditions (Figure 5F), since in this mutant *HAP43* is expressed under any of those conditions. Notably, the *Δhap43* mutant grows poorly under low iron conditions and the *Δsfu1* mutant grows poorly under high iron conditions, yet differences of the same strain in the presence and absence of tempol remained significant. Under high iron conditions addition of 0.5 mg/ml tempol led to a threefold increase of ATP quantification of revertant strains *HAP43*-R and *SFU1*-R compared to their controls without tempol (Figures 5E,F). Thus, it seems that under these conditions the disturbance of iron homeostasis mediated by mid concentrations of tempol is rather promoting metabolic activity than subverting it. At concentrations of 1 mg/ml of tempol this effect was abrogated again (Figures 5E,F). Taken together, this series of experiments demonstrates that iron homeostasis is disturbed by tempol and the effect is *HAP43*-dependent.

In addition, tempol treatment induced genes which were previously shown to be associated in either stress responses to or resistance against antifungal drug treatment. Cell wall genes *RBT5 and PGA7* involved in hemoglobin utilization and biofilm formation, respectively, were two of the most upregulated transcripts at both time points (Figure 4A, 30 min white bars/60 min dark bars). These genes are also regulated by Hap43p and interestingly have proposed roles in stress responses upon exposure to antifungal drugs, mainly azoles (Supplementary Table S4). Treatment with azoles fluconazole and ketoconazole affecting ergosterol synthesis induced *RBT5* and *PAG7* (Liu et al., 2005; Sosinska et al., 2008; Singh et al., 2011; Sorgo et al., 2011).

Genes involved in cell cycle processes, such as *MSH2* and *PMS1*, were also induced by tempol (Figure 4A). Mutants of *PMS1* or *MSH2*, both mismatch repair genes, have shown to render *C. albicans* more resistant against antifungal drugs (Legrand et al., 2007).

**FIGURE 6:**
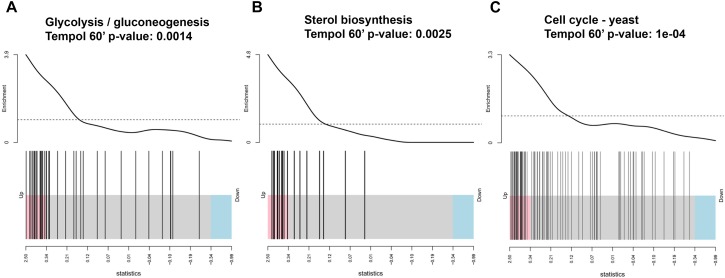
Tempol induces a metabolic stress response in *C. albicans* affecting **(A)** glucose/gluconeogenesis, **(B)** sterol biosynthesis, **(C)** cell cycle pathways as determined by KEGG pathway analyses of the differentially expressed genes and shown by barcode pilot function. Samples analyzed were treated for 60 min with 0.344 mg/ml tempol and compared to untreated samples.

To harness more information from transcriptional profiling we have performed KEGG pathway analyses of the differentially expressed genes (Figure 6). Tempol affected pathways with fundamental importance for fungal homeostasis, such as glycolysis and gluconeogenesis (Figure 6A and Supplementary Figure S4A), steroid biosynthesis (Figure 6B and Supplementary Figure S4B), and yeast cell cycle (Figure 6C and Supplementary Figure S4C). Glycolysis and gluconeogenesis are essential for survival of *C. albicans* both in commensal as well as pathogenic habitats. These pathways are the most common inducible pathways to metabolize glucose from external sources or to generate glucose from non-carbohydrate carbon substrates. *C. albicans* needs to control these pathways meticulously in order to thrive in different environments (Barelle et al., 2006). Other pathways involved in the production of building blocks for gluconeogenesis, such as amino acids biosynthetic and catabolic pathways (alanine/aspartate and tyrosine pathway), were altered by tempol treatment. In addition, genes involved in the steroid biosynthesis pathway, responsible for the production of the main fungal sterol ergosterol, were also significantly affected (Figure 6B). This is in good agreement with the GO term analysis where iron homeostasis and mitochondrial processes were influenced by tempol. Mitochondrial dysregulation is connected to membrane disturbance, since regulation of lipid homeostasis and the synthesis of membrane phospholipids depend on mitochondrial function (Shingu-Vazquez and Traven, 2011). A correlation to membrane dysfunction is further confirmed by tempol-induced deregulation of several genes which have been shown to be important for resistance against azole (Supplementary Table S4), a class of drugs which target ergosterol synthesis in fungi. However, we could not observe a short-term disturbance of mitochondrial membrane potential as measured using MitoTracker (Supplementary Figure S5). After 30 min of tempol treatment the mitochondrial membrane potential remained unchanged with increasing tempol concentrations (up to 1 mg/ml). Later time points did not lead to conclusive results, since *C. albicans* started to induce hyphal growth which interfered with the flow cytometric analysis. Further analyses are required to determine whether tempol disturbs the mitochondrial membrane potential at later time points. The observed mitochondrial dysregulation could be more indirect as a consequence of dysregulation of iron homeostasis (Figure 5).

Since the transcriptomic profile of tempol-treated *C. albicans* revealed a dysregulation of pathways related to glycolysis/gluconeogenesis and amino acid metabolism we analyzed metabolic footprints in growth media of *C. albicans* suspension cultures challenged with tempol compared to untreated controls. We focused on amino acid and carbohydrate metabolites. As we also observed significant changes in the sterol synthesis pathway, we used metabolic footprints of fluconazole-treated *C. albicans* cultures as an additional reference.

In total, 50 metabolites were identified and corrected by the ISs prior to statistical analysis. On average the relative standard deviation (RSD) of the 50 metabolites was 7.6 ± 6.4% in six QC samples and all RSDs were <30%, confirming the stability of the metabolomics experiment (Supplementary Figure S6).

**FIGURE 7:**
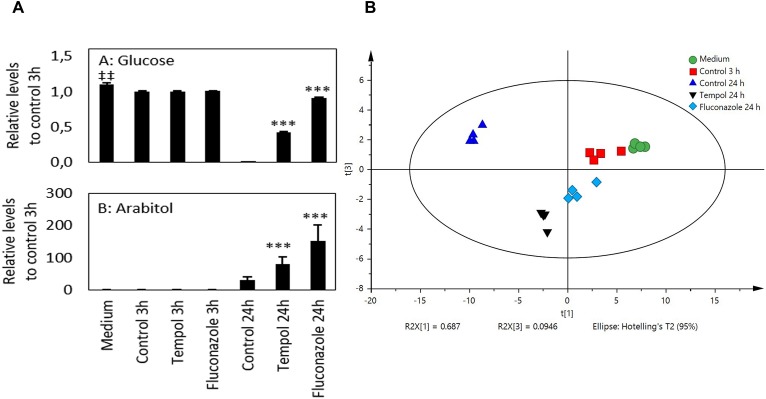
Tempol induces a metabolic stress response in *C. albicans* affecting mainly Krebs cycle metabolites. **(A)** Glucose uptake and D-arabitol excretion of *C. albicans* at different time points after treatment with fluconazole (2 μM equals 0.613 μg/ml) and tempol (2 mM equals 0.344 mg/ml). *P*-values were calculated against control samples of *C. albicans* at 3 and 24 h, respectively. ^‡⁣‡^*p* < 0.01 compared to 3 h control sample. ^∗∗∗^*p* < 0.001 compared to 24 h control sample. Two-sided Welch’s *t*-test (*n* = 4). **(B)** PCA score plot of 50 metabolites that were analyzed by GC–TOF–MS (*n* = 4); (R2X = 0.894). The treatment protocols for the different samples analyzed are described in the section “Materials and Methods”.

Glucose uptake was hindered by treatment with tempol and fluconazole compared to control samples at 24 h (Figure 7A). After 3 h, the levels of six metabolites were significantly lower and of two metabolites higher in samples treated with tempol (not significant after FDR adjustment). Fluconazole-treated samples showed similar results (three metabolite levels significantly higher and three metabolites lower compared to control samples). After FDR adjustment, only tryptophan concentration was significantly higher in *C. albicans* treated with fluconazole (Supplementary Figure S6). After 24 h the levels of 34 metabolites were significantly higher and of 8 metabolites lower in media of *C. albicans* treated with tempol (29/10 for fluconazole, respectively, FDR adjusted). The samples treated with tempol 24 h and control 24 h samples clustered together as well as all samples at 3 h. Fluconazole 24 h clusters closed with samples at 3 h confirming the effective alteration of the growth of *C. albicans* (Figure 7B and Supplementary Figure S7). A principal component analysis (PCA) was performed on autoscaled data. Three principal components (PCs) explained 89.4% of the variation in the data and the different conditions separate from each other, whereas the replicates grouped together (Figure 7B and Supplementary Figure S7). The first component explains 69% of the variation of the data and separates the different conditions. Among others, mainly metabolites that take part in the Krebs cycle, such as malic acid, succinic acid, α-ketoglutarate, pyruvic acid, and glucose contribute to PC1 (Supplementary Figures S7, S8A), confirming a dysregulation of glycolysis and respiration by tempol. PC3 separates untreated *C. albicans* samples from samples challenged with tempol or fluconazole. The latter correlated with higher levels of xylulose, arabitol, xylose, and ribitol (Figure 7A and Supplementary Figure S8B). PC3 contains stress response information of *C. albicans* to exogenous compounds. That is, metabolites that have a high contribution to PC3 are likely metabolites that *C. albicans* produces upon mounting a stress response which is induced by treatment with tempol or fluconazole. Taken together, the metabolomic analysis confirmed dysregulation of glycolysis/gluconeogenesis pathways and general stress responses in *C. albicans* which resembles antifungal agents targeting fungal membranes.

## Discussion

In the search for treatment options of invasive mycoses clinicians face limitations of both number of alternative substances to choose from and a very narrow selection of fungal targets which current antifungal agents attack. Academic groups seeking to understand the biology and virulence of fungal pathogens should harness new possibilities and take responsibility to find new approaches with more specific fungal targets without compromising the host and the microbiota. It is important to note that researchers at non-profit academic institutions cannot act in full as a pharmaceutical developer and launch a product from screen to selling. Deliverables from academia are, however, deep understanding of the biology of fungi and exploration of new ways how to exploit this knowledge. The scope of this study was to repurpose a pharmacologically active compound tested for other diseases in order to serve as an antifungal agent. Redox-cycling nitroxide tempol is a well-established radical scavenger tested to avoid radiation damage during treatment of cancer patients (Metz et al., 2004). Previous work has shown that targeting antioxidant pathways in fungal pathogens by pharmacological thioredoxin inhibition could prevent corneal infection (Leal et al., 2012). While testing tempol for anti-inflammatory potential (Hosseinzadeh et al., 2012), we observed its antifungal activity which we further investigated during this study. We present evidence for a considerable fungicidal effect on several *Candida* species with a transient effect on the fungal burden in an experimental model of systemic candidiasis. ICs (0.15–0.68 mg/ml) and MFC (0.5 mg/ml) of tempol for *C. albicans* might appear high. However, tempol has a promising safety profile with very low toxicity in humans, as revealed during application as topical treatment with a concentration exceeding 400 mM (equaling 68.9 mg/ml) (Metz et al., 2004). In addition, due to the high solubility in water considerably high *in vivo* concentrations may be reached. Systemic administration of tempol in mice resulted in high peak concentrations, such as in blood (8.1 mM equaling 1.4 mg/ml) or in kidneys (7.2 mM equaling 1.25 mg/ml) (Davis et al., 2011). Thus, *in vitro* efficacy concentrations determined in our study can be reached in vertebrate animals with no obvious toxic effects, allowing us to speculate that tempol or derivatives thereof could be applied as an antifungal agent to treat systemic infections in humans.

In order to find clues for the mode of action, we analyzed whole-genome transcriptomic responses of *C. albicans* to tempol using RNA-Seq. Upon tempol treatment, numerous of the differentially expressed genes have been previously shown to be involved in iron homeostasis or mitochondrial function. Both cellular processes are intertwined, since mitochondria depend on many iron-containing proteins and also harbor dedicated iron transporters (Xu et al., 2014). In accordance, 22 of these proteins are regulated by Hap43p (Supplementary Table S4 and Supplementary Figure S3), a central transcription factor for iron homeostasis. Susceptibility to tempol treatment was interestingly reduced in a Δ*hap43* knockout mutant strain, indicating that the Hap43p-mediated response toward tempol is rather leading to hazardous consequences in the fungal cell. By challenging *C. albicans* with tempol at high and low iron conditions, we confirmed that tempol treatment induced dysregulation of iron homeostasis hazardous to the fungal cells in dependence of transcription factor Hap43p (Figure 5).

An analysis of KEGG pathways and a metabolomics investigation identified dysregulation of genes involved in glycolysis and gluconeogenesis, indicative for increased energy production via glycolytic fermentation due to mitochondrial stress. In addition, mitochondrial function is important for membrane homeostasis via regulation of phospholipid and sterol synthesis (Shingu-Vazquez and Traven, 2011). KEGG pathway analysis revealed that tempol treatment disturbed sterol biosynthesis and induced general stress responses. As we could not observe short-term changes of mitochondrial membrane potential upon tempol treatment (Supplementary Figure S5) the tempol-mediated effects on gene expression related to mitochondrial dysregulation and membrane stress might be more indirect and a consequence of dysregulation of iron homeostasis which is also closely linked to mitochondrial function (Xu et al., 2014). Hence, our results suggest that tempol directly or indirectly interferes with pathways of iron homeostasis leading to a subsequent shift in energy metabolism, mitochondrial physiology, and membrane stress. Further target identification studies will be required to determine molecular target(s) within these affected pathways.

In general, metabolites are end products and mediators of cellular processes and thus metabolic footprinting can be used to depict stress response in yeast (Raamsdonk et al., 2001). Thereby, extracellular metabolites were measured in spend culture media of yeast overcoming the challenges of detecting intracellular metabolites such as their rapid turnover (Allen et al., 2003). Since we focused our analyses on carbohydrate- and amino acid-related metabolites we were not able to integrate the metabolomic data into the pathway analysis with transcriptomic profiling. Nevertheless, our analysis detected interferences of tempol with pathways related to glucose and alternative carbon source metabolism (Figures 6A–C and Supplementary Figure S4).

We decided to test *in vivo* efficacy of the compound in a murine model of systemic candidiasis. The dosing regimen, we used, was based on a detailed pharmacokinetic study (Davis et al., 2011). A caveat resulting from this investigation was the transient nature of the tempol-mediated reduction of fungal burden in the kidneys of *C. albicans*-infected mice. Tempol reduced the fungal burden in kidneys significantly at day 1 *p.i*. (Figure 2A), indicating that therapeutic doses of tempol may be achieved *in vivo*. The effect was, however, not maintained at day 3 p.i., despite daily doses were applied. We assume that due to the high solubility, tempol cannot be retained and is quickly removed via renal clearance which could lead to the transient effect of tempol in the murine model of systemic candidiasis. A pharmacological formulation of tempol could solve this problem. However, since we have not quantified renal clearance for this study, other possibilities for the brief antifungal effect need to be considered. It has previously been shown that tempol affects phagocyte activities *in vitro*, such as release of DNA traps and kinase activity important for ROS generation (Hosseinzadeh et al., 2012; Santos et al., 2018). As these effects directly hamper phagocyte antifungal activity, tempol treatment could lead to ineffective fungal clearance at later time points. Notably, besides transiently reducing the fungal burden, tempol treatment also increased blood-glucose concentration back to normal levels in infected mice. Sham-treated and infected mice were hypoglycemic correlating to the decreased wellbeing of animals (Figure 2B). For the presented *in vivo* tests, we received ethical permission for a maximum of 3 days (A12-13 and A80-14, Swedish Board of Agriculture) and the experiments needed to be terminated before any animals would succumb due to the lethal intravenous dose of *C. albicans*. Thus, we were not able to prolong treatment to assess long-term efficacy.

More remarkably, tempol could increase the immune response against *C. albicans* in human blood. Levels of IL-8, MCP-1, MIF, VEGF, and RANTES, all cytokines involved in leucocyte recruitment and activation (Filler et al., 1996; Dongari-Bagtzoglou and Kashleva, 2003; Stylianou et al., 2015) were specifically increased in tempol-treated blood infected with *C. albicans* (Figures 3A–E). Interestingly, fluconazole has also been shown to induce several of these cytokines during experimental candidiasis (Maneenil et al., 2015), suggesting that tempol shares properties of a common antifungal drug.

In conclusion, our study revealed that tempol has antifungal and immune-modulatory activities which resemble commonly used antimycotica. High solubility most probably determines transient *in vivo* efficacy which could be overcome by proper formulation of the compound. The low toxicity and good safety profile of tempol propose the compound as promising candidate for development of new therapy options, particularly, in regard to the common toxic side effects observed during current antifungal therapy.

## Materials and Methods

### Fungal Strains and Culture Conditions

*Candida albicans* wild-type strain SC5314 (Gillum et al., 1984), Δ*hap43* knockout strain CaHap43D2-1-8-1, Δ*hap43-HAP43* revertant strain CaHap43R3-1-5-2, *Δsfu1* knockout strain CaSfu1D1-1-3-1, *Δsfu1-SFU1* revertant strain CaSfu1R2-2-2-1 (Hsu et al., 2011), and *C. glabrata* ATCC 90030 were incubated in synthetic complete dropout medium (SC medium) + 2% glucose overnight at 30°C. A fresh subculture of *C. albicans* was inoculated in SC medium at 1 × 10^7^ cells/ml at 30°C for 4 h. Finally, for experiments, cell number was adjusted, according to the European Committee on Antimicrobial Susceptibility Testing-Antifungal Susceptibility Testing (EUCAST-AFST) guidelines, to ≥2 × 10^5^ cells/ml. Clinical isolates of *C. albicans* (CA40, CA75, CA79) and *C. glabrata* (CG67, CG71, CG77) from positive blood cultures originated from the strain collection of the Norrland University Hospital, Umeå, Sweden and are a kind gift of Dr. Margareta Granlund.

### Fungal Susceptibility Testing

Cell concentration and tempol (Sigma–Aldrich) microdilution were based on AFST guidelines for IC leading to 50 and 90% growth inhibition (IC_90_ and IC_50_) (Arendrup et al., 2012). After subculture 5 × 10^5^ cells/ml of *C. albicans* yeast cells were distributed in black 96-well/plates with clear-bottom containing RPMI 1640 medium supplemented with 10 mM HEPES (Thermo Fisher Scientific). Subsequently, tempol was added into 10 different concentrations (0.05–17 mg/ml) and incubated 24 h at 30°C.

The IC_90_ and IC_50_ were calculated from at least three biological replicates in triplicates using CellTiter-Glo luminescent cell viability kit (Promega) in a luminometer (Tecan InfiniteF200) (Stylianou et al., 2014). The antifungal activity of tempol was determined as a ratio of [(luminescence levels of tempol-challenged *C. albicans* cells)/(luminescence levels of 100% *C. albicans* growth)].

The minimum fungicidal concentration (MFC: 99.9% killing) was determined using spot-agar assay (Prasad et al., 2010). Challenged cells were transferred onto yeast peptone dextrose + 2% glucose (YEPD) agar plates using a multi-comb and incubated 24 h at 30°C.

For experiments under high iron and iron limiting conditions we followed the instructions of Hsu et al. (2011). Briefly, YEPD was considered a high iron medium and *C. albicans* strains from YEPD plates were cultured in YEPD liquid medium overnight at 30°C. After subculture 5 × 10^5^ cells/ml of *C. albicans* yeast cells were distributed in black 96-well/plates with clear-bottom containing YEPD liquid medium. To achieve low iron conditions *C. albicans* strains from a YEPD agar plate were subcultured overnight in YEPD containing 400 μM basophenanthrolinedisulfonate disodium salt (BPS; Sigma–Aldrich) to deplete iron storages. After subculture 5 × 10^5^ cells/ml of *C. albicans* yeast cells were distributed in black 96-well/plates with clear-bottom containing YEPD liquid medium containing 200 μM BPS to maintain iron-limiting conditions. Tempol was added at different concentrations as indicated and plates were incubated for 24 h at 37°C. In this context, we considered RPMI and SC media as norm-iron conditions with low, but sufficient iron concentration.

### Transcriptome Analyses of *C. albicans*

*Candida albicans*, subcultured as described, was incubated in RPMI 1640 medium at 37°C with or without tempol (2 mM equals 0.344 mg/ml) for 30 and 60 min. Cells were frozen as beads by dropping into liquid nitrogen. Disruption was carried out using a Mixer MillMM200 (RETSCH, Germany) with a shaking frequency of 30/s under cryo conditions. The resulting powder was resuspended in lysis buffer RLTplus (QIAGEN, Germany), supplemented with 0.01% v/v of β-mercaptoethanol. The extraction of total RNA was performed according to QIAGEN’s Mechanical Disruption Protocol for the isolation of total RNA from yeast, using the RNeasy Plus Mini Kit. The experiments were performed in duplicates. RNA quality and quantity was evaluated using an Agilent 2100 Bioanalyzer with RNA 6000 Nano Chips, following the manufacturer’s protocol.

cDNA library was generated with 300 ng of total RNA of each sample using Illumina’s TruSeq RNA Sample Prep Kit v2 according to manufacturer protocol. Quality and quantity were checked with Agilent 2100 Bioanalyzer using DNA 1000 Kit. Sequencing run was carried out on a HiSeq 2000 with single-end 60 bases long reads following the manufacturer’s instructions. In summary, for each sample between 15 and 20 million reads were generated.

Mapping was performed with NextGenMap (v 0.4.12) using default settings (Sedlazeck et al., 2013). We used sequence files from Assembly21^1^ and annotation files from Grumaz et al. (2013) as reference database. Gene quantification was calculated with a python script “rpkmforgenes.py” from the Sandberg laboratory^2^ at readcount and RPKM level [=reads per kilobase of exon model per million mapped reads, according to Mortazavi et al. (2008)] using uniquely mapped reads. However, differential gene expression profiling was carried out exclusively based on readcount quantification via DESeq2 package (version 1.8.2) developed for RNA-Seq data (Love et al., 2014).

### Pathway Analysis

KEGG pathway annotations (PMID: 10592173) were obtained via the KEGGREST R package for *C. albicans*. The respective gene identifiers from the RNA-seq experiment were mapped to KEGG nomenclature and assigned to 120 pathways (last accessed July 2018). We used the geneSetTest function from the limma package (Ritchie et al., 2015) to assess whether gene members of a given pathway differ significantly in their log2 expression fold changes relative to all other genes (outside of the pathway). Significant results (*p*-value < 0.05) were visualized by the barcode pilot function from the limma package.

### Metabolomics by GC–TOF–MS

*Candida albicans*, subcultured as described, was incubated in RPMI 1640 medium with or without tempol (2 mM equals 0.344 mg/ml) for 3 and 24 h. SAD fluconazole (2 μM) was used as control. The samples, including six quality control (QC) samples, were prepared as described previously with some modifications (A et al., 2005): Of the extraction mix 450 μl was added to 50 μl sample, vortexed for 5 min and incubated for 2 h at 4–8°C. Then, the mix was centrifuged (4°C, 14,000 rpm, 10 min) and 100 μl of the supernatant was evaporated to complete dryness (1.5–2 h). Subsequently, 30 μl of methoxyamine in pyridine was added, vortexed, and incubated at 70°C for 1 h followed by 15 h at room temperature. Prior to GC–TOF–MS analysis, 30 μl of *N*-methyl-*N*-trimethylsilyl-trifluoroacetamide + 1% trimethylchlorosilane and 30 μl of heptane containing 15 ng/μl methyl stearate were added, vortexed, and incubated at 70°C for 60 min. Subsequently, 1 μl of the sample was injected splitless bz an CTC Combi Pal XT Duo (CTC Analytics AG, Switzerland) into an Agilent 7890A gas chromatograph equipped with a 30 m × 0.25 mm i.d. fused-silica capillary column with a chemically bonded 0.25-μm DB-MS UI stationary phase (J&W Scientific, Folsom, CA, United States). Further GC settings were as follows: injector temperature: 260°C, purge flow: 20 ml/min after 75 s, carrier gas: Helium, carrier flow: 1 ml/min, column temperature was held at 70°C for 2 min, then increased by 20°C/min to 320°C, final temperature was held for 4 min. The column was introduced into the ion source of a Pegasus III TOF–MS (Leco Corp., St Joseph, MI, United States). The TOF–MS settings were as follows: transfer line temperature: 250°C, ion source temperature: 200°C, ionization energy: 70 eV, acquired mass range: *m*/*z* 50 to *m*/*z* 800 with a scan rate of 20 Hz, acceleration voltage: turned on after solvent delay of 290 s.

### Assessment of Mitochondrial Membrane Potential

The mitochondrial membrane potential of *C. albicans* cells was determined using MitoTracker Green FM (Thermo Fisher Scientific) and subsequent flow cytometry analysis. Exponentially grown *C. albicans* cells were washed twice with PBS and treated or not with different concentrations of tempol for 30 min in RPMI at 37°C. MitoTracker was added to *C. albicans* suspensions at a final concentration of 100 nM and the cultures were further incubated at 37°C for 30 min in dark. Cells were then washed twice with PBS to remove residual dye. Fluorescence was quantitatively assessed using flow cytometry (BD Accuri^TM^ C6) using 10^6^ *C. albicans* cells.

### Blood Infection and Plasma Collection for Cytokine Assay

Heparinized blood isolated from three healthy individuals was infected under cell culture conditions with 2 × 10^5^ cells/ml of *C. albicans* in triplicates overnight at 37°C. Infected samples were treated with 0.516 mg/ml of tempol or treated with vehicle only. After incubation overnight, plasma was separated from cells and debris at 300 g at room temperature and stored at −80°C. Uninfected blood samples and uninfected blood samples treated with tempol (0.516 mg/ml) served as controls.

The release of cytokines was assessed using the Bio-Plex human cytokine 27-plex and 21-plex panel (Bio-Plex Pro #M50-0KCAF0Y and #MF0-005KMII, BIORAD). Positive hits were confirmed using a customized Bio-Plex human cytokine 10-plex. Briefly, antibody-coated beads specific to the tested cytokines were incubated with the plasma samples. Subsequently, PE-streptavidin conjugated detection antibody was added and the fluorescent intensity was measured from the Bio-Plex 200 assay reader (Bio-Rad Laboratories AB). The cytokine concentration was based on the supplied standard samples provided by the manufacturer.

### Systemic *C. albicans* Infection in Mice

*In vivo* efficacy of tempol was tested in a systemic mouse candidiasis model. Mice used were 6–8 weeks old female C57BL/6 (in average mice weighed approximately 22 g at start of experiment). *C. albicans* cells were grown overnight at 30°C in SC medium. Infection of mice was performed i.v. with 200 μl from a 2.6 × 10^6^ *C. albicans* cells/ml suspension by injection into the tail of 20 mice. Subsequently, 10 out of 20 mice were i.p. injected with 100 μl of 40 mg/ml tempol in PBS while the remaining 10 mice were injected with 100 μl PBS. Additionally, five uninfected mice were injected with tempol to monitor potential compound-related effects.

Temperature and weight were controlled for all mice daily during the first 48 h. After that time point mice were checked every 8 h. Five mice from each infected group were anesthetized (Ketalar 11.25 mg/Dormitor 0.1 mg per mouse) at day 1 p.i. and another group of 5 at day 3 p.i. Blood was collected by heart puncture for biochemistry testing and analyzed using the VetScan Critical Care Plus reagent rotor (Abaxis). Uninfected controls were also analyzed to determine the background baseline. Mice were sacrificed and the kidneys dissected and weighted. Kidneys were homogenized and plated on YEPD plates and the number of growing colonies was used to determine fungal burden. Fungal load was expressed as a measure of the number of *C. albicans* CFU per gram of kidney. The whole experiment was split into two separate experiment periods and hence each group of five animals adds up to a total of 10 mice per group for the evaluation of results.

### Statistical Analysis

Inhibitory concentration (AZL > 50%)/IC (AMB > 90%) and *n* = 5(3) analyses and determination were performed based on ATP levels as previously described from the following equation% IC_AZL_ > _50__% AMB_ > _90__%_ = 100 − (value_sample_/value_growth control_) * 100 using Microsoft Office Excel 2007 (Stylianou et al., 2014). MFC *n* = 3(4) determination was qualitatively assessed at the concentration of drug that caused no notable growth on agar plates to which fungal suspensions were transferred after incubation with different tempol concentrations.

For transcriptional analyses using RNA-Seq we only considered adjusted *p*-values below 0.05 according to DESeq2 package to determine the significance of differences in expression profiles as described previously (Storey and Tibshirani, 2003).

Metabolomics data *n* = 4(4). Additionally, a pool was prepared from aliquots of each sample to provide QC samples. The QC samples were randomly prepared and analyzed within the metabolomics batch to investigate the stability and reproducibility of the analysis. GC–TOF–MS analysis, data processing (correction to IS), and metabolite identification were performed as described previously (Kauppi et al., 2016). Excel 2010 (Microsoft, Albuquerque, NM, United States) and Matlab R2014a (MathWorks, Natick, MA, United States) were used for univariate statistical analysis, and PCA was done in SIMCA (Simca 14, Umetrics AB, Umeå, Sweden).

Data for cytokine profiling *n* = 3 (3) evaluated with one-way ANOVA with a Tukey post-test.

Systemic candidiasis in mice was performed at two different experimental days in groups of five mice with a total of 50 mice. Data used for CFU and biochemistry blood tests were analyzed using a Mann–Whitney *U*-test.

The statistical calculations and analyses were performed using GraphPad Prism Software 6.05 (GraphPad Software, La Jolla, CA, United States) and for all analyses, *p*-values < 0.05 were considered statistically significant.

## Data Availability

The datasets generated for this study can be found in the European Nucleotide Archive where the RNA-Seq data are available. The accession number is PRJEB31969.

## Ethics Statement

Blood from healthy donors was obtained from the Department of Laboratory Medicine at Umeå University Hospital. Samples were taken with previous written consent on an entirely voluntary basis with no financial compensation. Blood samples were forwarded anonymously to not be traced back and considered as the donation of average healthy donors. The Department complies in full with European bioethical laws. The animal experiment reached its endpoint after 3 days and follow animal ethical guidelines stated in permission A12-13 and A80-14 from the Swedish Board of Agriculture and Umeå Ethical Committee for Animals.

## Author Contributions

CU, AHo, MS, and JL conceived and designed the experiments. MS, AHo, JL, DM, AHä, SH, and CG performed the experiments. AHo, MS, JL, DM, CG, AHä, CU, and CD analyzed the data. KS, AJ, and CD contributed reagents, materials, and analysis tools. CU, JL, MS, and AHo wrote the manuscript.

## Conflict of Interest Statement

The authors declare that the research was conducted in the absence of any commercial or financial relationships that could be construed as a potential conflict of interest.

## Footnotes

1 http://www.candidagenome.org/download/sequence/C_albicans_SC5314/Assembly21/

2 http://sandberg.cmb.ki.se/rnaseq
