# Supplementary material for: Stable Redox-Cycling Nitroxide Tempol Has Antifungal and Immune-Modulatory Properties
Source: Front Microbiol. 2019 Aug 20;10:1843. doi: 10.3389/fmicb.2019.01843 (PMC6710993; doi:10.3389/fmicb.2019.01843)
Supplement: Supplementary file 1 [file Table_1.pdf]

Table S1. ***In vitro*** **activity of tempol against** ***C. glabrata*** **ATCC 90030 assessed by 3 different methods.**

IC and MFC of tempol against *C. glabrata*			
Tempol	**ATP**	**OD_{530} (nm)**	**Agar-spot assay**
Tested Concentration range (mg/ml)	**0.05 - 17**	**0.05 - 17**	**-**
IC_{90} [a]	**1.4**	**1.4**	**-**
IC_{50} [b]	**0.3**	**0.5**	**-**
MFC[c]	**-**	**-**	**1.4**

[a,b] Inhibitory concentration (IC) was determined as the lowest concentration of tempol that inhibited fungal growth at ≥90% IC_{90}[a] or ≥50% IC_{50} [b]; Data from three independent experiments. [c]Minimum fungicidal concentration (MFC) was determined as the lowest concentration of tempol that killed at least 99.9% of the initial inoculum. Data from three independent experiments.
