# Supplementary material for: Stable Redox-Cycling Nitroxide Tempol Has Antifungal and Immune-Modulatory Properties
Source: Front Microbiol. 2019 Aug 20;10:1843. doi: 10.3389/fmicb.2019.01843 (PMC6710993; doi:10.3389/fmicb.2019.01843)
Supplement: Supplementary file 2 [file Table_2.pdf]

Table S2. ***In vitro*** **activity of tempol against** ***C. albicans*** **and** ***C. glabrata*** **clinical isolates using ATP quantification.**

**Susceptibility of clinical *Candida* isolates in response to tempol**<br>**Tested concentration range (mg/ml) 0.05 - 17**			
C. albicans clinical strains			
	CA40	CA75	CA79
IC_{90} [a]	1.26	1.84	1.85
IC_{50} [b]	0.17	0.33	0.40
C. glabrata clinical strains			
	CG67	CG77	CG71
IC_{90} [a]	1.92	2.53	3.63
IC_{50} [a]	0.26	0.40	0.46

[a,b] Inhibitory concentration (IC) was determined as the lowest concentration of tempol that inhibited fungal growth at ≥90% [a]IC_{90}[a] or ≥50% IC_{50} [b]; Data from three independent experiments. [c]Minimum fungicidal concentration (MFC) was determined as the lowest concentration of tempol that killed at least 99.9% of the initial inoculum. Data from three independent experiments.
