# Supplementary material for: Stable Redox-Cycling Nitroxide Tempol Has Antifungal and Immune-Modulatory Properties
Source: Front Microbiol. 2019 Aug 20;10:1843. doi: 10.3389/fmicb.2019.01843 (PMC6710993; doi:10.3389/fmicb.2019.01843)
Supplement: Supplementary file 4 [file Table_4.pdf]

Table S4. Name and function of the differentially expressed *C. albicans* genes affected upon tempol challenge.

Gene	Expression / function	Deregulated by other antifungal compounds	Relevant references
PGA7	GPI-linked hyphal surface antigen important for iron assimilation	Azoles	(Liu et al., 2005) (Synnott et al., 2010)
RBT5	Rbt5p is glycosylphosphatidylinositol (GPI)-modified cell wall protein. Expression is induced by iron limitation	Ketoconazole, ciclopirox olamine, caspofungin and amphotericin B	(Kronstad et al., 2013) (Lee et al., 2005)
CSA1	Surface antigen on elongating hyphae and buds	Ciclopirox	(Sigle et al., 2005a)
FET34	Multicopper ferroxidase; induced by low iron	Ciclopirox olamine and ketoconazole	(Rogers and Barker, 2002;Liu et al., 2005)
FRP1	Ferric reductase	Fluconazole and ciclopirox	(Copping et al., 2005)
FTR1	High-affinity iron permease	Amphotericin B, caspofungin, ciclopirox,	(Niewerth et al., 2003;Lee et al., 2005;Sigle et al., 2005b)
CDC21	Putative thymidylate synthase	Fluconazole	(Liu et al., 2005)
DUT1	dUTP pyrophosphatase		
RNR1	Ribonucleotide reductase large subunit; induced in low iron		
MSH2	Putative DNA mismatch repair factor	Flucytosine	(Liu et al., 2005)
PDS5	Putative protein with a predicted role in establishment and maintenance of sister chromatid condensation and cohesion		
PMS1	Putative DNA mismatch repair factor		(Legrand et al., 2007)
POL30	Proliferating cell nuclear antigen (PCNA)	Flucytosine	(Liu et al., 2005)
RAD51	Protein involved in homologous recombination and DNA repair		(Liu et al., 2005)
RAD57	Putative DNA recombination and repair protein		
RFA1	Putative DNA replication factor A		
TUB1	Alpha-tubulin		
ADE 5,7			
ADE2	Phosphoribosylaminoimidazole carboxylase activity		
ADE6	5-Phosphoribosylformyl glycinamidine synthetase		
GCV2	Glycine decarboxylase P subunit; protein of glycine catabolism		
GCV1	Putative T subunit of glycine decarboxylase		
MSS116	Putative DEAD-box protein		

orf19.5701	Role in DNA replication initiation		
RPO41	Putative mitochondrial RNA polymerase		
orf19.2631	Subunit of Elongator complex		
orf19.5038	Predicted tRNA (guanine) methyltransferase activity		
orf19.512	S. cerevisiae ortholog is essential and is required for biogenesis of the small ribosomal subunit		
PUS7	Pseudouridine synthase;		
