# Supplementary figures and images for: Stable Redox-Cycling Nitroxide Tempol Has Antifungal and Immune-Modulatory Properties
Source: Front Microbiol. 2019 Aug 20;10:1843. doi: 10.3389/fmicb.2019.01843 (PMC6710993; doi:10.3389/fmicb.2019.01843)

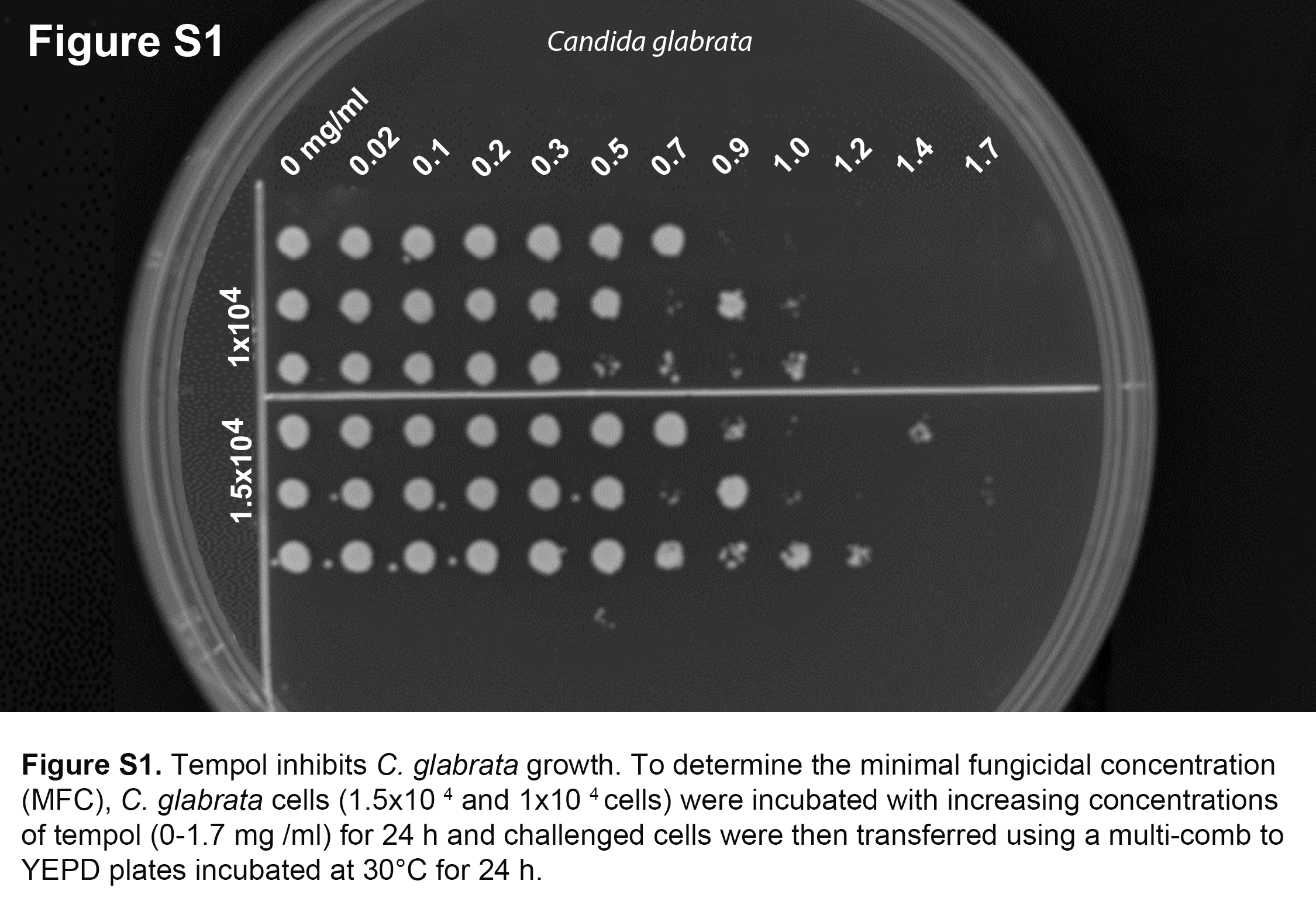

Supplement: Supplementary file 5 [file Image_1.TIF]

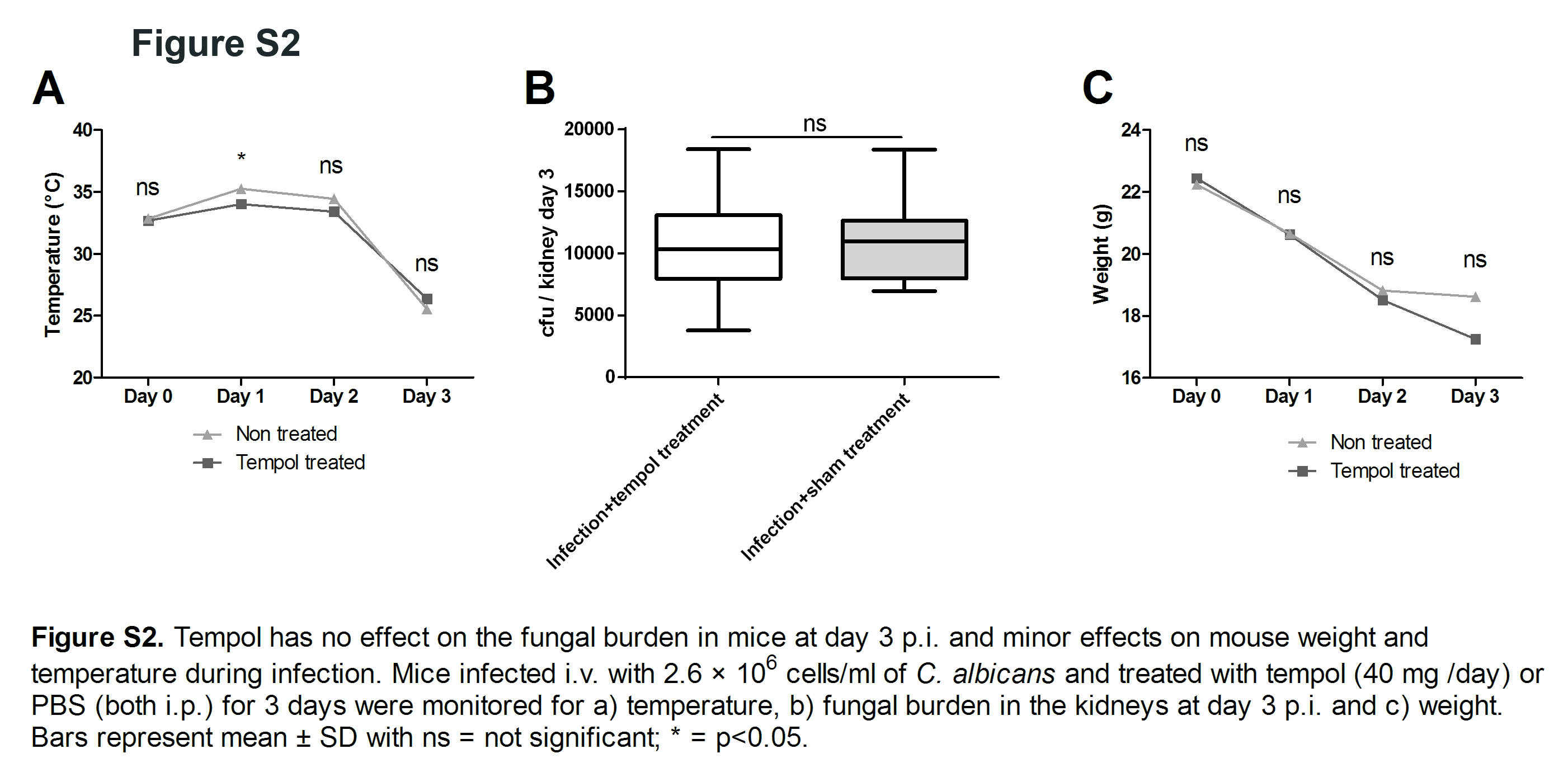

Supplement: Supplementary file 6 [file Image_2.TIF]

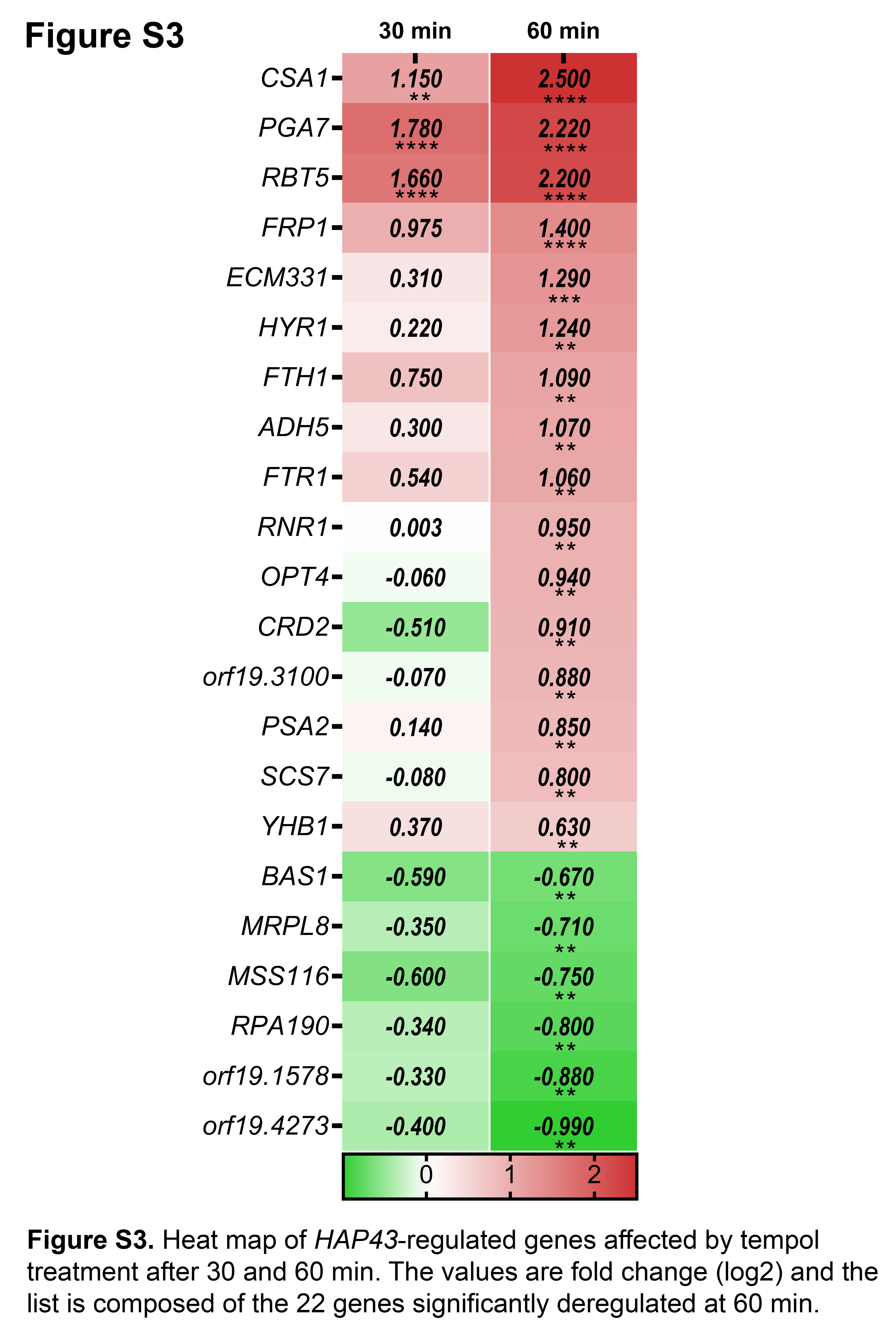

Supplement: Supplementary file 7 [file Image_3.TIF]

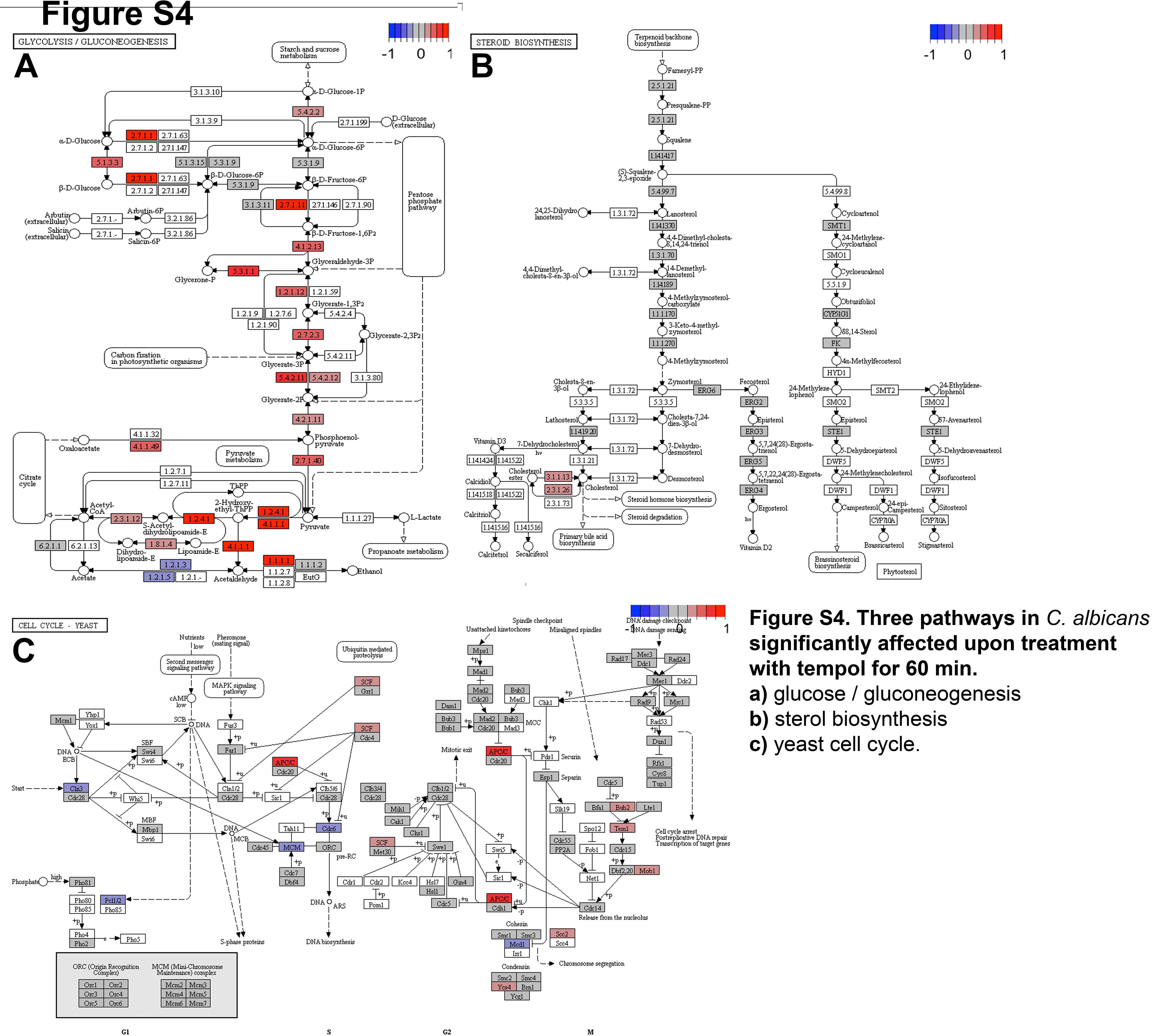

Supplement: Supplementary file 8 [file Image_4.TIF]

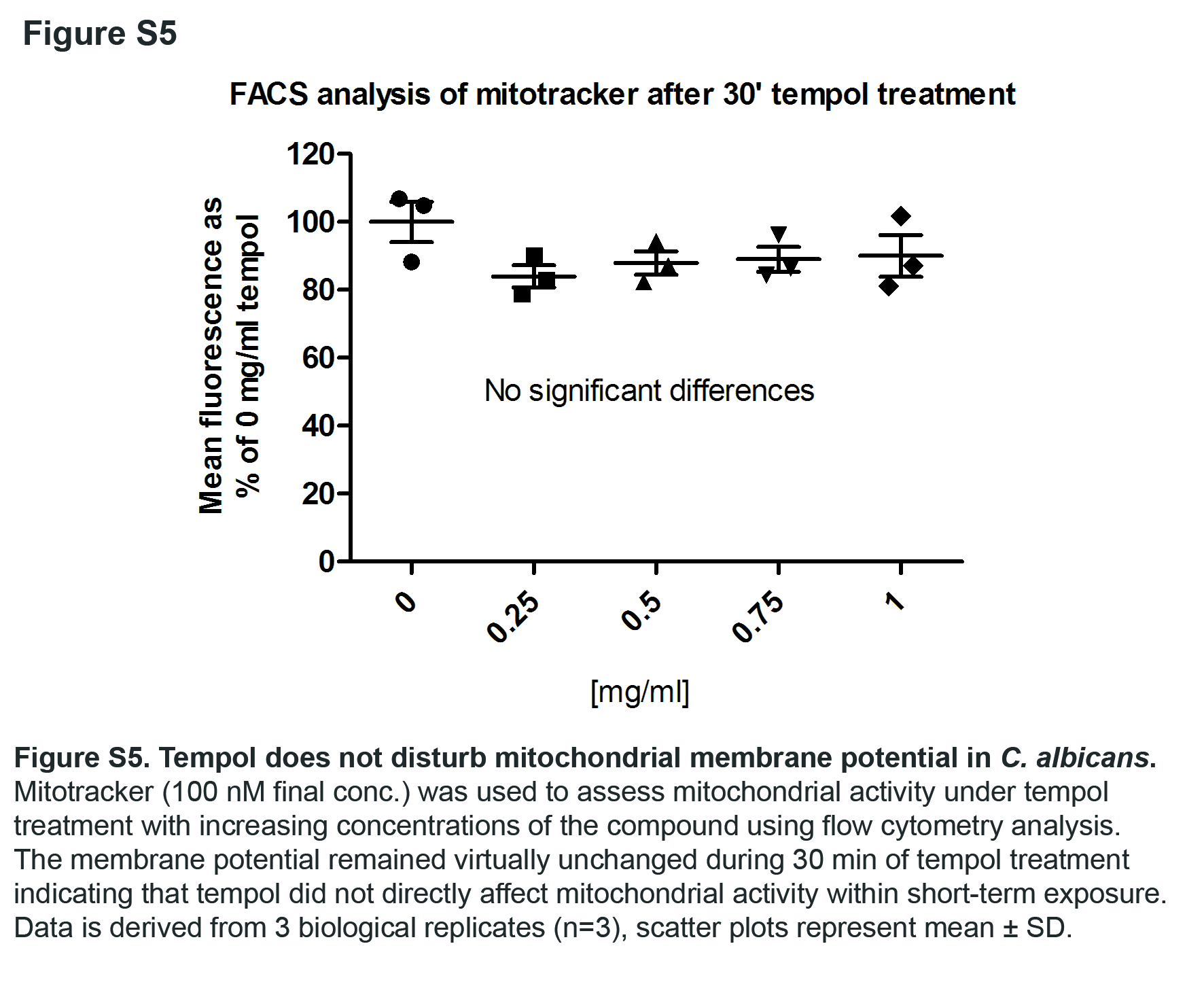

Supplement: Supplementary file 9 [file Image_5.TIF]

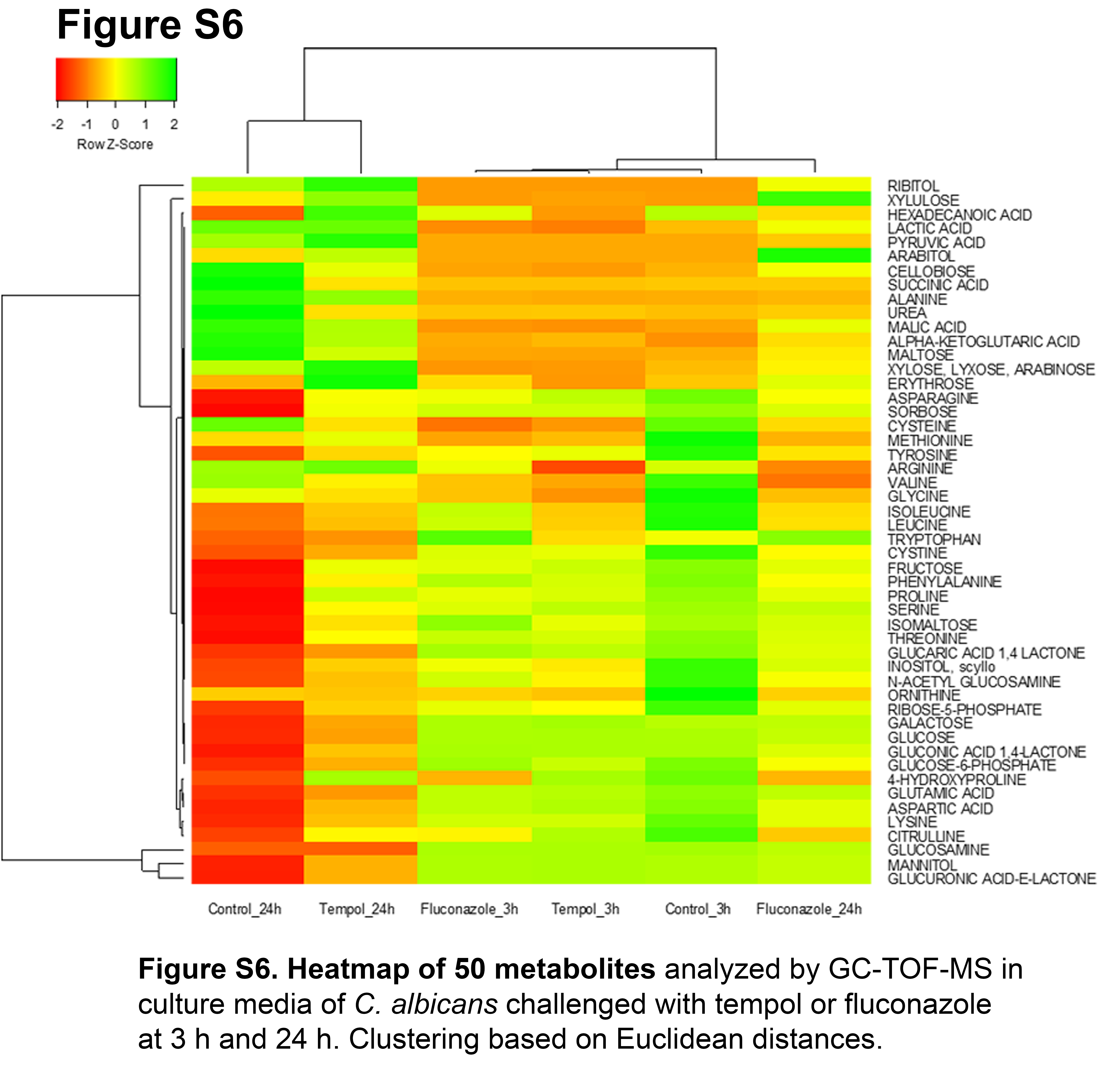

Supplement: Supplementary file 10 [file Image_6.TIF]

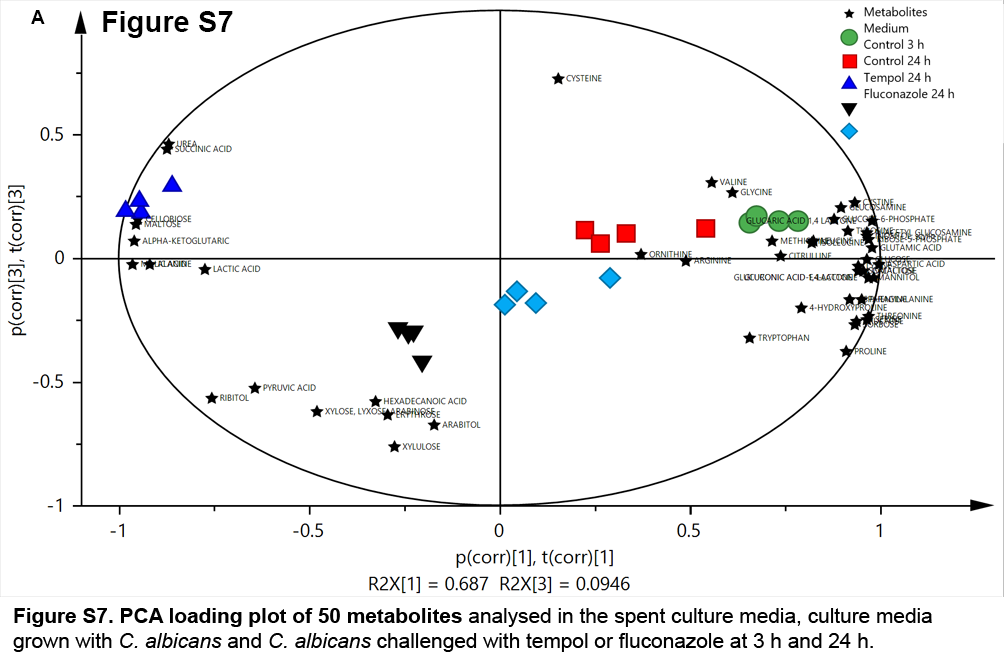

Supplement: Supplementary file 11 [file Image_7.tif]

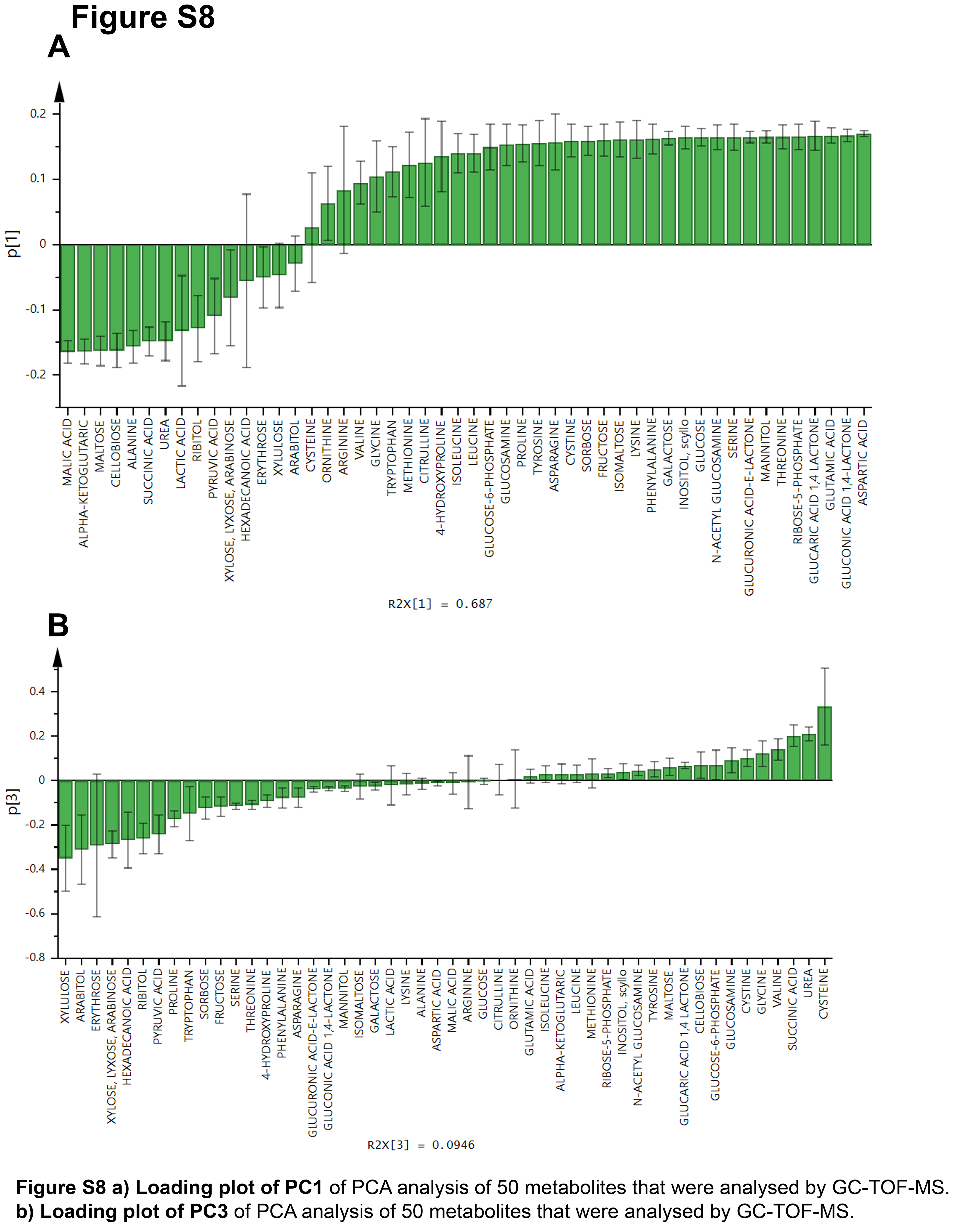

Supplement: Supplementary file 12 [file Image_8.tif]
